# Economic evaluations of colorectal cancer screening: A systematic review and quality assessment

**DOI:** 10.1016/j.clinsp.2023.100203

**Published:** 2023-04-25

**Authors:** Marcela Castro Ramos, Julio Augusto de Lima Passone, Ana Carolina de Freitas Lopes, Adriana Vaz Safatle-Ribeiro, Ulysses Ribeiro Júnior, Patrícia Coelho de Soárez

**Affiliations:** a Departamento de Medicina Preventiva, Faculdade de Medicina, Universidade de São Paulo (FMUSP), São Paulo, SP, Brazil; b Faculdade de Saúde Pública (FSP), Universidade de São Paulo (USP), São Paulo, SP, Brazil; c Departamento de Gastroenterologia, Faculdade de Medicina, Universidade de São Paulo (FMUSP), São Paulo, SP, Brazil

**Keywords:** Colorectal neoplasms, Cost-benefit analysis, Systematic review

## Abstract

Colorectal Cancer (CRC) is the third most common type of cancer worldwide and ranks second in mortality. Screening programs for early detection and treatment have been implemented in several countries. Economic evaluations are an important tool to support decision-making about reimbursement and coverage decisions in health systems and, therefore, to support efficient resource allocation. The article aims to review the up-to-date evidence on economic evaluations of CRC screening strategies. MEDLINE, EMBASE, Web of Science, SCOPUS, SciELO, Lilacs, CRD databases, and lists of references were reviewed to identify relevant literature regarding full economic evaluations of CRC screening in asymptomatic average-risk individuals over 40 years old. Searches were conducted with no restriction to language, setting, or date. Qualitative syntheses described CRC screening strategies and comparators (baseline context), study designs, key parameter inputs and incremental cost-effectiveness ratios. Seventy-nine articles were included. Most of the studies were from high-income countries and a third-party payer perspective. Markov models were predominantly used, although microsimulation has been increasingly adopted in the last 15 years. The authors found 88 different screening strategies for CRC, which differed in the type of technique, the interval of screening, and the strategy, i.e., isolated or combined. The annual fecal immunochemical test was the most predominant screening strategy. All studies reported cost-effective results in their scenarios compared to no screening scenarios. One-quarter of the publications reported cost-saving results. It is still necessary to develop future economic evaluations in Low- and Middle-Income Countries (LMICs), which account for the high burden of disease.

## Highlights

• Colorectal Cancer (CRC) screening programs reduced the incidence and mortality of CRC.
• CRC screening strategies were cost-effective or cost-saving, mainly in high-income countries.
• There is a need to develop economic evaluation studies for CRC screening in low- and middle-income countries.

## Introduction

Cancer is the second leading cause of death and represents a major public health issue worldwide. Despite scientific advances, there were ten million deaths from cancer in 2020 and an increasing disease burden due to the growth and aging of the population, as well as other environmental risk factors [1].

Colorectal Cancer (CRC), also called colon and rectal cancer, refers to the type of malignant tumors that affect the large intestine. According to estimates from the International Agency for Research on Cancer (IARC), it is the third most common type of cancer worldwide, with 1.9 million new cases and 935,000 deaths in the past year, ranking second in mortality rates [1]. Historically, CRC has been a broad issue in high-income countries, yet increasing incidence rates have been noted in developing countries, such as Eastern Europe, southeastern and south-central Asia, and South America [2,3]. Brazil has revealed the largest incidence worldwide in recent years, followed by Costa Rica and the Philippines, where the mortality rates were higher [2,4].

CRC is a heterogeneous disease related to lifestyle, environmental and genetic factors [5]. In developing countries, there have been increasing trends in new cases associated with Western influences and changes in lifestyle, diet, and other known risk factors [6]. In addition, age is a relevant risk factor for CRC development. United States incidence data have increased twice each successive decade for patients between the ages of 40 and 80 years old [7]. The high turnover rate of the intestinal epithelium promotes a hotspot for malignant occurrences and adenomatous polyp development. Adenomas are the most frequent precursors (precancerous lesions) that progress into CRC [8]. In general, the progression of the adenoma to an invasive neoplasm stage occurs slowly and usually takes several years [9,10].

Although aging decreases relative survival, the stage at diagnosis is still the most important prognostic factor. When CRC is detected at early stages (39% of cases), there is a 90% 5-year relative survival rate [11]. Over the past decades, the prognosis of CRC has improved, with better survival rates in high-income countries than in Low- and Middle-Income Countries (LMICs). This heterogeneity may be explained by the degree of surveillance, screening programs, treatment availability, and accessibility in those countries. Early detection strategies, including population-based screening programs, are an important component to reduce the morbidity and mortality of many types of cancer [6].

Considering its high incidence, long preclinical phase, recognizable and treatable precursors, high cost of treatment associated with health services, and the correlation of mortality with the diagnostic stage, CRC has become a good option for screening. While most types of screening aim to detect the early stages of cancer (secondary prevention), there are modalities, such as cervical cancer and CRC screening programs, that detect precancerous lesions. This type of screening impacts not only mortality but also incidence (primary prevention program) [8].

The economic burden related to cancer treatment in society is substantial. Expenditures on treatment, as well as indirect costs related to a lack of productivity and disability, strongly impact health systems in all countries. Indeed, some studies conducted from the perspective of LMICs confirmed the high economic burden of CRC [12,13].

Economic evaluations are valuable tools to advocate decision-making toward the choice of health technologies in public health and, therefore, support efficient resource allocation. Over the years, the authors have seen an increasing tendency of economic evaluation studies for CCR screening to report cost-effective results for a varied number of screening strategies in different baseline scenarios.

Systematic reviews of economic evaluations in health care improve the decision-making processes and the quality of future studies. These studies provide a comprehensive summary of efficiency results and explicitly identify the methodological issues of the reviewed studies [14]. Systematic reviews of economic evaluations of CRC screening strategies have been published with some restrictions on language, setting, screening strategy, or outcomes [15], [16], [17]. To support a CRC screening economic evaluation for program development in LMICs, the authors reviewed the evidence available on economic evaluations published to date to identify CRC screening strategies, the decision models used to measure costs and health benefits, and the incremental cost-effectiveness of modeled screening strategies.

## Methods

### Study design

This study provides a systematic review of economic evaluation studies of CRC screening strategies in asymptomatic average-risk populations and is registered in the International Prospective Register of Systematic Reviews (PROSPERO) database (CRD42018103739) [18].

The protocol was developed based on the guidance of the center for Reviews and Dissemination (CRD) for undertaking reviews in health care [19] and was reported accordingly to the Preferred Reporting Items for Systematic Reviews and Meta-analyses statement for protocols (PRISMA-P) [20].

### Objectives

The review provides detailed data on the existing evidence for CRC screening economic evaluations to justify and support these evaluations and program development in LMICs.

### Eligibility criteria

#### Type of studies

Inclusion criteria: Full economic evaluation studies were included regardless of publication period, language, or setting. Trial-based and model-based economic evaluations were eligible for inclusion.

Exclusion criteria: Outcomes and cost evaluation studies, systematic reviews, and partial economic evaluations were not considered. Abstracts, editorials, letters, posters, conference communications, methodological discussion articles, and reviews were also excluded from this review. However, it is important to highlight that although some reviews were not eligible for inclusion, their references list was used for relevant study detection and inclusion.

#### Participants/population

The population for this research comprised economic evaluation studies in which there was intervention (CRC screening) provided to asymptomatic individuals over 40 years old with no increased risk of CRC development.

#### Type of intervention/comparator

Any CRC screening tests were eligible for inclusion, and the comparator(s) comprised the baseline context and no-screening scenarios.

#### Type of outcome measures

There were no study outcome restrictions applied to this protocol, as the purpose of this review was also to identify which outcomes in relation to decision model construction were reported in selected studies. Nonetheless, the following relevant primary outcomes may be measures of cost-effectiveness: Quality-Adjusted Life Years (QALYs), Disability-Adjusted Life Years (DALYs), Incremental Cost-Effectiveness Ratio (ICER), cost-benefit ratio, and net benefits, as well as life years saved, cases avoided, deaths prevented and others.

### Search strategy

The following electronic bibliographic databases were searched to identify relevant literature concerning economic evaluations of CRC screening: MEDLINE (via PubMed), EMBASE, Web of Science, SCOPUS, Scientific Electronic Library Online (SciELO), Regional Virtual Health Library, Center for Reviews and Dissemination (CRD) Databases (Database of Abstracts of Reviews of Effects (DARE), the National Health Service Economic Evaluation Databases (NHS EED), and the Health Technology Assessment Database (HTA). Additionally, reference lists were manually reviewed to detect further studies possibly unaccounted for in all the included and relevant articles identified during the search. All databases were screened with no restriction for publication period or language, and before the final analyses, searches were performed for further study retrieval.

The exploration strategy construction purposefully covered this review's three major eligibility criteria domains (colorectal cancer, screening strategies, and full economic evaluations) by searching all relevant terms and combining them using Boolean operators. Term combinations within each domain used “OR” and were later combined using “AND”. To expand the sensitivity analysis for each investigation, there was a combination of free text, indexing terms, database-specific subject headings/vocabulary (e.g., MeSH and EMTREE), and other keyword variants. A proficient librarian in economic evaluations’ systematic reviews reviewed the exploration strategies of this protocol, which are detailed in Online Resource 1.

### Study selection

Initial searches retrieved citations (through December 2020) that were uploaded, deduplicated, and managed in Rayyan software [21]. The detected duplicate articles were manually removed prior to review initiation. Three independent reviewers (MCR, JALP and ACFL) with expertise in economic evaluation studies and systematic reviews screened the identified citations’ titles and abstracts according to the eligibility criteria. This step confirmed and assessed the reports for potential eligibility. Another reviewer (PCS) was included in case of any disagreement on study inclusion.

The PRISMA diagram illustrates studies that were not relevant for the review and the summarized exclusion reasons.

### Data extraction

Two experienced reviewers (MCR and JALP) conducted the full papers’ data extraction according to a predefined extraction form on Microsoft Excel. A third reviewer (PCS) concluded any discrepancies. A pilot test before the official data extraction guaranteed both reviewers’ alignment regarding specific information extraction in each category by using a random sample of five studies. The following topics summarize the extracted data: (1) Publications’ general characteristics, including first the author's, publication year and journal; (2) Study characteristics, such as economic evaluation type, setting, population, interventions, comparators, and evaluated outcomes; and (3) Model characteristics, including the type of model, time horizon, currency and reference year, discount rate, cost types, incremental cost-effectiveness ratio, willingness to pay values and sensitivity analysis details (key parameter inputs, assumptions and their impact in the analysis).

### Strategy for data synthesis and reporting quality assessment

A narrative synthesis was provided, and the data were summarized in tables to structure the general characteristics. The assessment of the report quality for the included studies was conducted according to the CHEERS (Consolidated Health Economic Evaluation Reporting Standards) checklist from the International Society of Pharmacoeconomics and Outcomes Research (ISPOR) taskforce [22].

Two reviewers (MCR and JALP) independently appraised the study methods across the checklist, and a third reviewer (PCS) made the final decision for any conflict.

### Statistical analysis

Summary statistics were reported as frequency and percentage of reports for main characteristics of colorectal cancer screening economic evaluations, and frequency and percentage of CRC screening strategies and incremental cost-effectiveness ratios. Statistical analyses involved the use of SPSS v.25.

## Results

### Search results

**Fig. 1:**
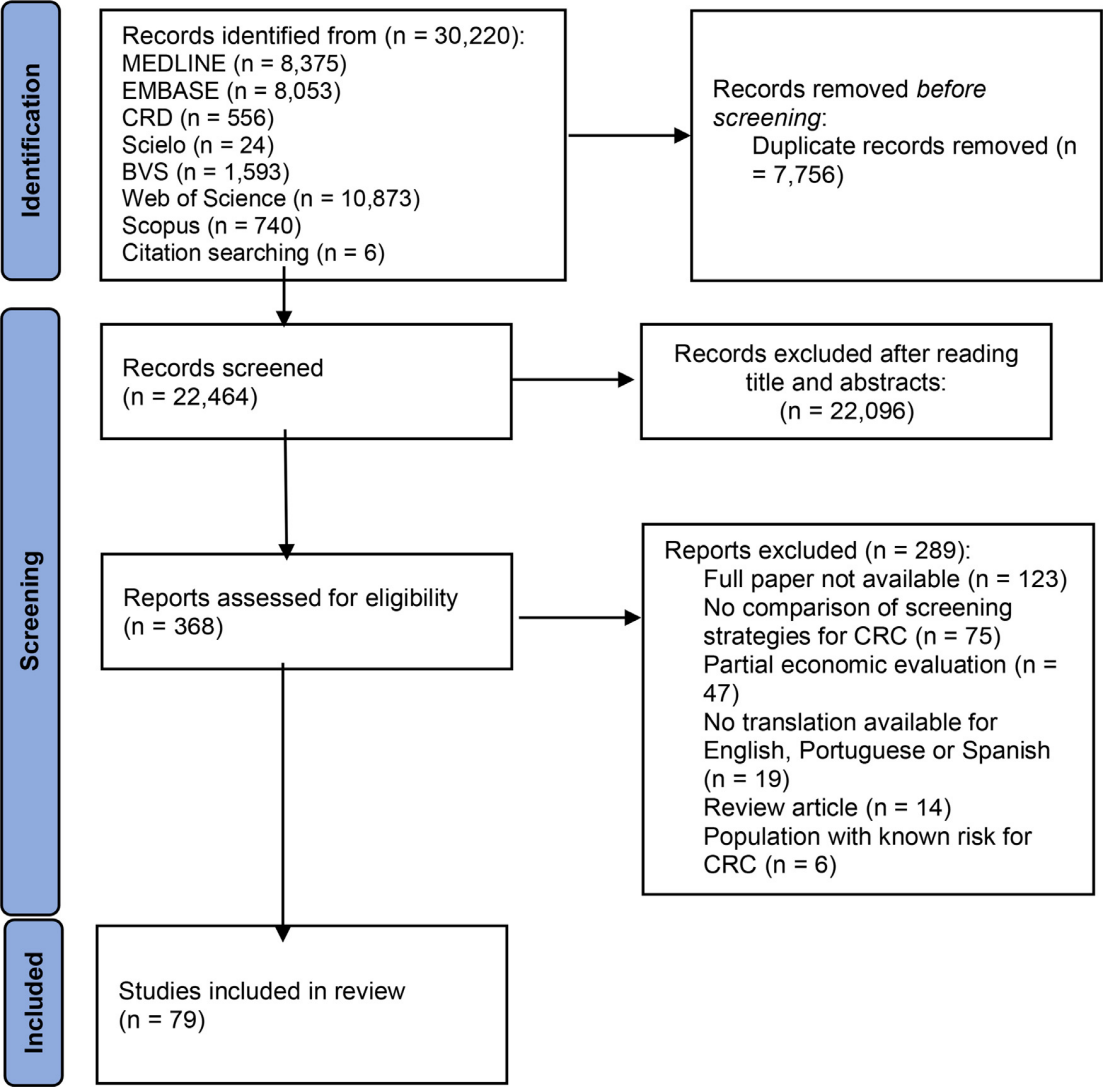
PRISMA diagram for study selection.

The initial research on the described databases retrieved 30,214 records in addition to six records identified through a reference list review, comprising 30,220 records (Fig. 1). There were 368 publications considered relevant for full-text screening after duplicate removal and title and abstract reading. Finally, 79 full economic evaluations on CRC screening were included based on the eligibility criteria (Supplementary Table 1).

### Main study characteristics

There was an increase in publications within the research period from 1991 to 2020, including a marked increase in the 2000s and a greater volume in the last decade (Table 1, and Supplementary Table 2). Most articles and studied populations were from North America and Europe, accounting for more than 70% of publications. The Asian population was included in 14% of the publications, followed by Oceania (8%) and South America (4%). Based on the World Bank classification, most studies account for high-income countries (91%), whereas upper-middle-income countries (8%) and lower-middle-income countries (1%) have little representativity.

**Table 1:** Main characteristics of colorectal cancer screening economic evaluations.

**Characteristics**	**n (%)**
**Countries**	
High-income	72 (91)
Upper middle-income	6 (8)
Lower middle-income	1 (1)
**Economic evaluation study**	
Cost-effectiveness analysis	54 (68.4)
Cost-utility analysis	25 (31.6)
**Model type**	
Markov	54 (68.4)
Microsimulation	17 (21.5)
Decision tree	1 (1.3)
Not specified	7 (8.8)
**Perspective**	
Third-party payer	58 (73.4)
Societal	13 (16.4)
Third-party payer and society	2 (2.6)
Not specified	6 (7.6)
**Time horizon**	
Lifetime	48 (60.7)
10 to 50 years	25 (31.7)
80 to 100 years	3 (3.8)
Not specified	3 (3.8)

The most frequent studies were cost-effectiveness analyses (68.4%) describing outcomes including mortality, incidence, number of deaths avoided, new case detection, Life Years Gained (LYG), or years of life lost. The cost-utility studies represented 31.6% of the total sample, and outcomes were represented by QALY.

The usual model type was the Markov model (68.4%). Since the 2000s, microsimulation models have represented 17.9% of the total, and in the past decade, there has been an increase in their use compared to the usage of Markov models. Notably, microsimulation models were only used to represent populations in the United States ( *n*  = 8), Netherlands ( *n*  = 3), Australia ( *n*  = 2), Canada ( *n*  = 1), France ( *n*  = 1), and the Basque country ( *n*  = 1). In 7.6% ( *n*  = 6) of the publications, the model and programming used were not specified or described.

The third-party payer perspective was predominantly adopted (73.4%), while in 16.4%, the societal perspective was considered. In 7.6% of the publications, the authors did not specify their choice. Finally, 2.6% of the publications considered both the society and the third-party payer perspective. In contrast, indirect costs were included in only 3% of the studies (productivity loss). All publications comprised direct costs (screening test, CRC treatment, colonoscopy complications, and colonoscopy cost), and 25% also added nonmedical direct costs (campaign organization, invitation to screening, screening kit delivery, transportation, and accommodation).

A lifetime horizon was applied in 48 (61%) studies. The other publications considered cohorts with a follow-up period between 10‒80 years, as described in Table 1, and Supplementary Table 2. In three studies, the time horizon description used in the analyses was unclear.

Regarding the discount rate, 57 studies (72%) considered 3% for both costs and outcomes; 5 studies considered 5%; 4 studies, 3.5%; 2 studies, 4%; one study, 3% (costs) and 1.5% (effectiveness); and 3 studies did not report the discount rates modeled.

### Population characteristics

For population characteristics, three age categories were predominant, resulting in 60% of the total: 50 to 80 years old (25.3%), 50 to 74 years old (17.7%), and 50 to 75 years old (16.5%).

Most studies ( *n*  = 57; 72.1%) adopted 50 years old as the starting age of screening. Other starting ages identified were 65 years old ( *n*  = 7; 8.9%), 40 years old ( *n*  = 6; 7.6%), 55 years old ( *n*  = 5; 5.1%), 60 years old ( *n*  = 3; 3.8%), and 58 years old ( *n*  = 1; 1.3%).

Regarding the upper limit age of screening, studies ranged from 60 to 100 years old. Most cohorts ended screening at 80 years old ( *n*  = 24; 30.4%), 75 years old ( *n*  = 18; 22.8%), and 74 years old ( *n*  = 16 studies; 20.2%). In three publications, the authors did not inform the upper age limit for screening. Details of the age ranges are presented in Supplementary Table 2.

During the publication eligibility screening phase, there were some studies in which the main objective was to determine the optimal age of CRC screening, and these studies were not included in this analysis. As the present study's goal was to include only full economic evaluations comparing different screening strategies, the authors excluded studies in which a comparison was made only between starting and upper limit ages, with the same screening tests evaluated in all scenarios.

Another essential parameter within the CRC screening context is the adherence rate. A high disparity was observed, varying from 29% to 100%, depending on the baseline scenario, type of screening test, and source of information considered when designing the model. Some authors established 100% in the base case but tested this parameter during the sensitivity analysis to better understand how screening adherence in the general population affects the cost-effectiveness analysis. Some studies considered different screening adherences depending on the screening strategy, which was frequently divided into blood-testing, stool-based testing, or imaging testing.

### Screening strategies for CCR

The following data refer to the primary screening strategy in each scenario. It is known that a colonoscopy must confirm all positive results from other noninvasive screening tests when a polypectomy if needed is also performed.

The authors identified 88 different screening strategies, differing from single or combined test techniques and screening intervals (age dependent or not). Single tests accounted for 87% of the screening strategies modeled in all studies, with imaging testing (45%) and stool-based tests (41%) being the most frequent, followed by blood tests (1%). The latter first appeared in 2013. A colonoscopy conducted every 10 years (14%), an annual FIT (8%), an annual gFOBT (7%), a biennial FIT (7%), a sigmoidoscopy every 5 years (6%), a biannual gFOBT (5%) and a CT every 10 years (4%) represent half of all screening strategies evaluated in the included studies.

In contrast, most combined strategies were stool-based testing in addition to imaging testing (13%). An annual gFOBT or FIT in addition to a sigmoidoscopy every 5 or 10 years were the most common combined strategies. One study modeled combined stool and blood-based tests, leveraging the screening strategy for those who were underscreened, defined as a population eligible for screening for which no past colonoscopy or FOBT had been performed in the previous 4 years.

The great majority of studies (94%) adopted a no-screening scenario as the main comparator, whereas in five studies (6%), the comparator was gFOBT.

### CRC screening cost-effectiveness

**Fig. 2:**
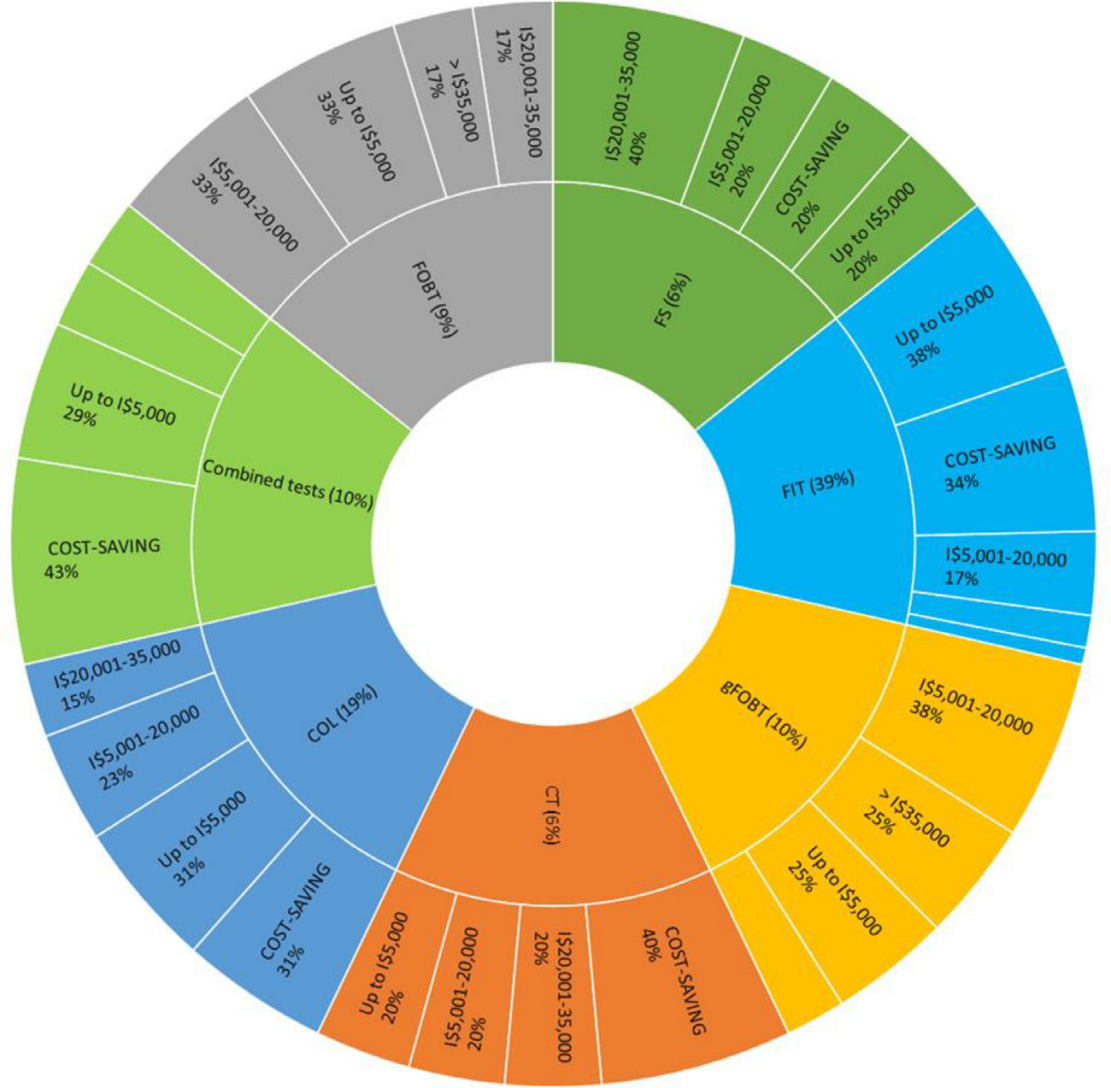
Colorectal cancer screening Incremental Cost-Effectiveness Ratios (ICERs) results. COL, Colonoscopy; CT, Computed Tomography; FIT, Fecal Immunochemical Test; FOBT, Fecal Occult Blood Test; FS, Flexible Sigmoidoscopy; Combined tests (When more than one type of test was used). *All ICERs were adjusted by local inflation to 2020 and then converted in International dollars (I$) using the Organization for Economic Co-operation and Development (OECD) Purchasing Power Parity conversion rates.

Incremental Cost-Effectiveness Ratios (ICERs) for each CRC screening strategy are presented in Fig. 2 and Supplementary Table 3. ICERs were adjusted for 2020 as the reference year and then converted into international dollars (Purchasing Power Parities) using the Organization for Economic Co-operation and Development (OECD) reference. Some measures were calculated in LYG, QALY, or both. The authors found great variation in incremental costs, which can be attributed to the modeled scenario, cost assumptions, and even the adherence rate adopted in the baseline scenario. Even though some studies did not state the Cost-Effectiveness Threshold (CET) in their reports, all authors concluded that compared to no screening, at least one CRC screening strategy in average-risk asymptomatic people over 40 years was cost-effective.

#### No screening comparator

Seventy-four economic evaluations (94%) adopted the no-screening scenario as the main comparator to evaluate the cost-effectiveness of a diversified number of strategies. These studies reported that one or more CRC screening strategies were cost-effective compared to no screening. FIT was the test most frequently reported as dominant over other evaluated strategies or over no screening in 35% of economic evaluations, followed by gFOBT (11%). In 11%, the FOBT technique was not reported. A colonoscopy was dominant in 21% of the scenarios, as detailed in Fig. 2 and Supplementary Table 3.

In addition, CRC screening was demonstrated to be cost-saving in 21% of economic evaluations, meaning that the screening program in the average-risk population would be more effective and less expensive in the modeled scenarios. One study was conducted from a low-middle income country perspective in Ukraine, and all screening strategies were cost-saving compared to no screening (annual FOBT, annual FOBT plus sigmoidoscopy every 5 years and colonoscopy every 10 years), whereas the other results were from high-income countries.

Other studies reported the following CRC screening strategies provided cost-saving results compared to no screening: colonoscopy only once, colonoscopy every 10 years, sigmoidoscopy only once, sigmoidoscopy every 5 years, CT every 10 years, annual gFOBT, FIT twice, annual FIT, biennial FIT, annual gFOBT plus sigmoidoscopy every 5 years, annual FIT plus sigmoidoscopy every 5 years, annual FIT plus colonoscopy at age 66, and biennial FIT plus colonoscopy every 5 or 10 years.

#### gFOBT comparator

Five studies (6%) used the gFOBT as the main comparator. In two studies, a CT every 10 years [28] and an annual FIT [31] were cost-saving compared to an annual gFOBT.

Two studies concluded that for CRC screening in average-risk individuals aged 50‒74 years in France, a biennial FIT was dominant compared to a biennial gFOBT. In contrast, one study concluded that a CT every 5 years was dominant among other evaluated strategies (FIT and CT).

### Sensitivity analyses

All studies conducted sensitivity analyses to verify which parameters affected the ICER the most; except for a single publication whose analysis was not reported (Supplementary Table 3). In one publication, the authors did not report which parameters affected the results. No sensitive parameters were identified after analysis in five studies. The adherence rate, costs, and accuracy of the screening tests were the parameters that most influenced ICERs.

### Reporting quality assessment

**Fig. 3:**
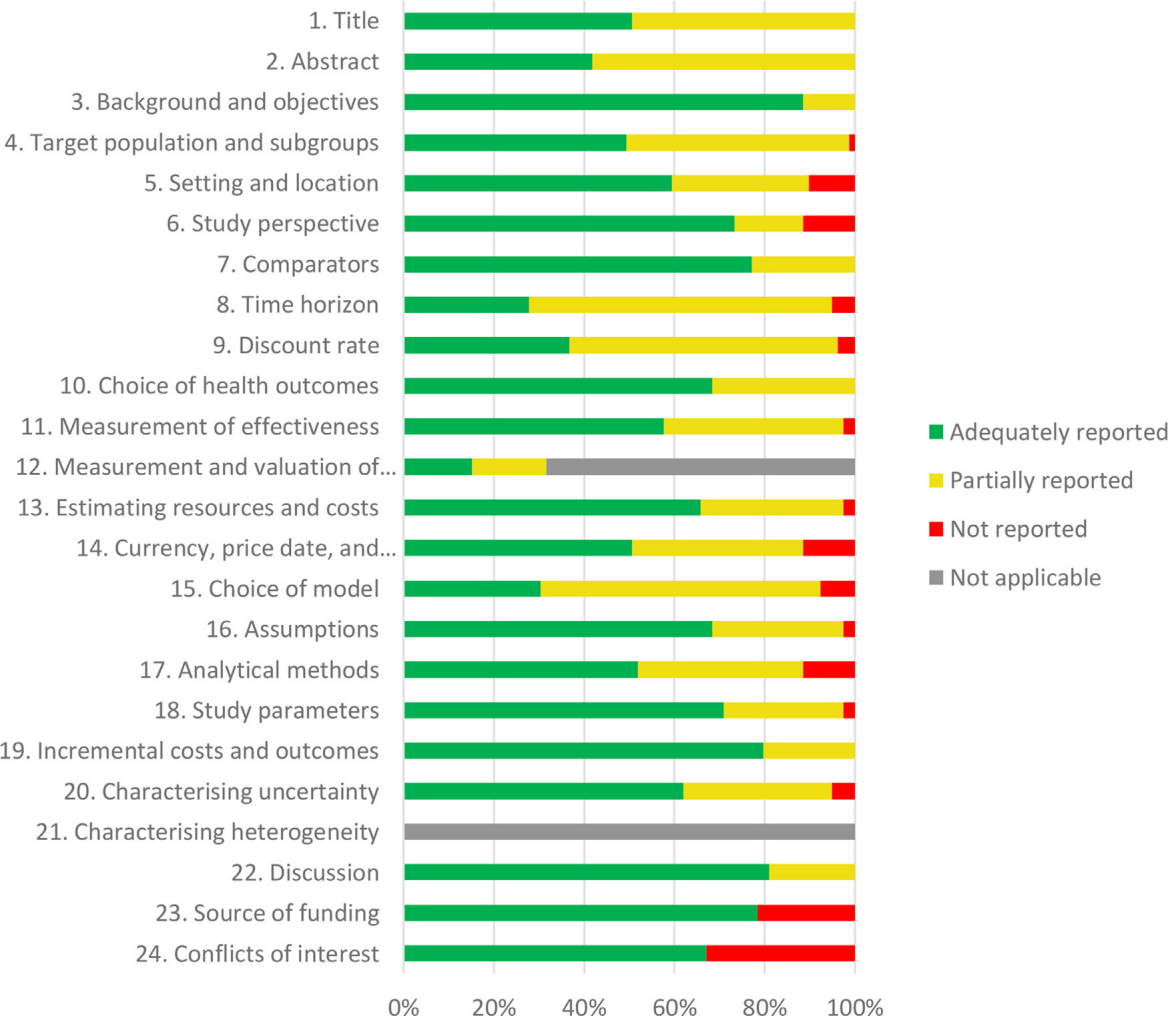
Report quality assessment by CHEERS checklist of all included economic evaluations of CRC screening.

Overall, as assessed by the CHEERS checklist, there was a historical improvement in economic evaluation reports over time. In general, the items adequately reported (≥ 70%) were the following: background and objectives, study perspective, comparators, study parameters, incremental costs and outcomes, discussion, and source of funding. In contrast, the items with less adequate reporting (≤ 40%) were time horizon, discount rate, and model choice (Fig. 3). The individual CHEERS assessment is presented in Online Resource 2.

## Discussion

This systematic review comprehensively identified CRC screening economic evaluation studies in the average-risk population worldwide. In this research, the authors found 79 full economic evaluations on CCR screening in the average-risk population over 40 years old. The first publication's date was in 1991, and there was an increase in this research subject in subsequent decades. Indeed, it is well known that based on their effectiveness and cost-effectiveness, population-based programs for CRC screening are available in several countries, mainly high-income countries.

The authors have seen an increasing tendency in making health decisions based not only on the efficacy and safety of new technologies but also on their effectiveness, efficiency, and budget impact. Hence, economic evaluations’ rising value in regard to the adoption of new technologies and the elaboration of national public policies has been highly noted. In fact, in some countries, economic evaluations are mandatory by many Health Technology Assessment (HTA) agencies for new technology adoption. This rationale is based on good management and allocation of health resources.

LMICs account for the higher burden of several diseases, and resource allocation is a great challenge that these places must tackle despite budget limitations [23]. However, a greater number of economic evaluations are performed in high-income countries [24,25]. This review brought to our knowledge that this is also true for CRC screening, and despite the high number of full economic evaluations on this subject, they mainly refer to high-income countries, in which population-based screening is generally well established. Although tendencies and findings can support and guide decisions, they are very unlikely to be generalizable to all countries.

There are several population-based CRC screening options currently available, and they are evolving rapidly. In fact, 88 different strategies were identified, demonstrating several combinations and possibilities that could be evaluated in Fig. 2 and Supplementary Table 3. The most common strategies were FIT, gFOBT, CT, sigmoidoscopy, colonoscopy, and fecal blood DNA testing, which can be either used in isolation or combined in different screening intervals. There are many variables to be considered, for example, previous preparation needed, risk of adverse events, cost, and frequency. In addition, the screening strategies will also impact health services differently, as some strategies require previous preparation, specialized personnel, and the use of physical space inside health institutions, such as colonoscopy and computed tomography [26], [27]. Therefore, economic evaluations are essential to draw each country's scenario and to support local decision-makers.

Inexpensive tests currently available, such as stool-based screening options, proved to be cost-effective and even cost-saving in many scenarios compared to no screening. The annual FIT was the dominant strategy in most studies and reported cost-saving results. Thus, for LMICs where infrastructure is limited (e.g., screening centers or facilities, qualified staff), it may be a good option for further assessment by local economic evaluations Fig. 2 and Supplementary Table 3.

The Markov model was predominantly used to represent the natural history of CRC and the cost-effectiveness of screening, followed by microsimulation models. There are validated models of both types in the literature for CRC screening, including critical appraisals of them [28]. Markov models, as static models, usually use yearly transition probabilities and focus on cohort proportions. One key implication of this could be the potential overestimating or underestimating of disease progression, therefore impacting cost-effectiveness results. In contrast, microsimulation can support individual-based models and allows time-dependent transitions’ incorporation across probabilities in a CRC progression, demonstrating increased modeling flexibility.

The increased use of microsimulation models in the 2000s for CRC representation can be a result of large initiatives in both Canada and the United States that allowed collaborations to develop health microsimulation models in the early 1990s and 2000, respectively. Notably, the degree of complexity and limited source data might also explain its slow adoption. Microsimulation models are particularly useful in the context of CRC screening, i.e., the screening of cancer with a prolonged natural history and heterogeneity, as they integrate several health determinants, states, and transitions between them. Such models may also be useful to measure costs and effects in different subgroups of individuals and for new tests. Although microsimulation models might be the best choice for this type of evaluation, all mathematical modeling has an inherent limitation, namely, the oversimplification of a real-life context. Model validation and uncertainty assessment of parameters, outcomes, and model structure are essential for finding robustness and addressing limitations regardless of the type [29]. In fact, only up to 30% of the included studies adequately reported the choice of model or its structure in full papers. Smith et al [30]. concluded that the absence of relevant information about model validity may be related to the journal word limit when publishing results, as most of the evaluated models were indeed validated after contacting the authors. In addition, the authors strongly recommend engagement with decision-makers during model building to leverage the evidence generated, its applicability, and its usefulness. Probabilistic sensitivity analysis is also fundamental to understanding the extent to which parameters affect the modeling and results.

Most studies chose 50 years as the ideal age to start screening in the baseline scenario. Four models that considered a start age below 50 years appeared in 2016, except for two Japanese studies (Supplementary Table 2). This may be related to the population's life expectancy, and opportunistic screening started in 1992 for individuals ≥ 40 years old in Japan [31].

It is well known that the adoption of national policies for CCR screening has had an impact on this type of cancer epidemiology, resulting in a reduction or stabilization of new cases, mortality, and morbidity in recent years for individuals ≥ 50 years [6]. However, in recent years, several studies have pointed to an increase in CRC incidence in the population ≤ 50 years, which has been seen mainly in high-income countries [6,[32], [33], [34], [35], [36], [37]]. This may be related to a higher prevalence and distribution of risk factors (obesity, alcohol intake, smoking, high-fat diet, low-fiber diet, sedentary lifestyle) in the younger generations, particularly those born close to the 1990s. Other factors were also attributed, such as the use of antibiotics in childhood [34] and the association of other diseases as cofactors, such as type 2 diabetes mellitus [38,39].

Observational studies have shown that screening strategies have also been effective in adults ≤ 50 years [40], [41], [42]. Despite these findings, the incidence remains higher in individuals over 50, and a screening program for all individuals (≤ 50 years) would not be cost-effective. However, Azad et al [43] concluded that the screening of individuals ≥ 40 years old proved to be cost-effective compared with a screening inclusion limited to people ≥ 50 years old, with a greater gain in QALYs and lower costs provided by sigmoidoscopy or FIT. Similarly, other studies using microsimulation models demonstrated additional gains in LYG, with the optimization of screening achieved by expanding the start age to asymptomatic individuals ≥ 45 years [44,45]. These results supported the extension to include people ≥ 40 years in CRC screening guidelines in some countries [46]. Future economic evaluations should acknowledge these findings and consider evaluating an extension of the optimal age range to include individuals ≥ 40 years to facilitate a better local assessment. One of the advantages of economic evaluations is the flexibility of applying all these factors to the model for further evaluation, where the resources that would be of most value to screening program coverage in each country could be determined.

Regarding the upper limit age for screening, the guidelines generally recommend individualized decisions for screening between 75 and 85 years and complete stopping after 85 years [46]. The degree of uncertainty regarding the benefits of screening in people over 75 years old is based on a low-value screening rational, which considers screening to be unlikely to bring additional benefits in terms of quality or years of life. The main aspects to be considered in deciding to conduct CRC screening in older individuals with normal results prior to colonoscopy are (1) Increased risk of adverse events and complications associated with colonoscopy at advanced ages, which can also be fatal for this group (e.g., perforation, bleeding, cardiovascular or pulmonary complications); (2) Unnecessary discomfort from the procedure; and (3) Anxiety generated by positive FOBT results [47,48]. In fact, the effectiveness of colonoscopy for individuals 75–79 years was deemed to be lower, and hospitalization due to adverse events doubled for individuals in this age group compared to individuals 70–74 years old [49]. Hence, diversified and individualized strategies for people over 75 should be proposed, such as risk factor stratification considering family history, physical condition, and patient preferences, to better determine the advantages of CRC screening offering and its benefit for this group.

Compared to the other published reviews, the present work represents an advance because it was not restricted by screening strategy, the model used, types of outcomes, region, date, or language. In this way, the authors were able to comprehensively understand how economic evaluations were performed worldwide and their main findings. This highlights relevant information that justifies the need for economic evaluation studies for CRC screening in other settings, such as developing countries.

This review found results similar to those of previous reviews [15], [16], [17], although the methodology somewhat differed regarding the location and scope of the review, resulting in a larger inclusion of studies.

Notably, the report quality of both titles and abstracts was not satisfactory (inadequate in approximately 50% of cases). Most titles failed to identify the publication as an economic evaluation by not using adequate terms, such as “cost-effectiveness analysis” or similar language and did not include all interventions being compared. The present search strategy was designed to capture different words used distinctively as indexed terms that relate to economic evaluations. However, issues may arise if there are no economic evaluation standardization filters across databases, and divergences may occur when conducting systematic reviews.

This review has some limitations. First, the authors did not perform a critical appraisal of the models used in each economic evaluation to represent the natural history of CRC and screening intervention because of the great number of publications included. Thus, future studies are needed to better understand how the design of studies impacts the results. Second, the authors did not present specific data regarding the sensitivity or specificity of stool-based tests because most publications lack this information or searches would have to be performed in gray literature. Last, one of the main purposes of the economic evaluation is to support and inform health decisions. Despite many studies published in recent years, it is difficult to generalize the results from this review to other locations, especially for LMICs in which their representativity in this analysis is extremely low.

## Conclusion

This review highlighted the need to develop further economic evaluation studies for CRC screening in developing countries. All included studies demonstrated cost-effective results mainly in high-income countries for most adopted CRC screening strategies evaluated against no screening scenarios, and approximately one quarter were cost-saving. CRC is still one of the most common and fatal tumor types. Policymakers should acknowledge that this type of screening can prevent the disease, reduce its mortality and, consequently, may provide savings in health expenditures, as previously demonstrated in many economic evaluations. Therefore, it should be considered a priority in public health and policymaking. More studies from the LMIC perspective are needed to better understand the ways in which CRC screening can be of value and the main barriers that should be considered when developing economic evaluations and screening programs for these countries.

## Authors’ contributions

Marcela Castro Ramos: Data curation, formal analysis, investigation, methodology, validation, writing - original draft.

Julio Augusto de Lima Passone: Data curation, formal analysis, investigation, methodology, validation, writing - original draft.

Ana Carolina de Freitas Lopes: Data curation, formal analysis, investigation, methodology, validation, writing - original draft.

Adriana Vaz Safatle-Ribeiro: Formal analysis, writing - review & editing.

Ulysses Ribeiro Júnior: Formal analysis, writing - review & editing.

Patrícia Coelho de Soárez: Conceptualization, data curation, formal analysis, investigation, methodology, validation, writing - review & editing.

## Funding

This study was supported with grants from the National Institute of Science and Technology for Health Technology Assessment (IATS) – CNPq/Brazil No. 465518–2014, with no role in the design of the study and collection, analysis, and interpretation of data and in writing the manuscript. de Soárez, Patricia Coelho is the recipient of a grant from the CNPq/Brazil No. 302268/2019-7.

## Conflicts of interest

The authors declare no conflicts of interest.

## Appendix Supplementary materials

Image, application 1
