# Supplementary material for: Economic evaluations of colorectal cancer screening: A systematic review and quality assessment
Source: Clinics (Sao Paulo). 2023 Apr 25;78:100203. doi: 10.1016/j.clinsp.2023.100203 (PMC10182269; doi:10.1016/j.clinsp.2023.100203)
Supplement: Supplementary file 1 [file mmc1.docx]

**CLINICS-D-22-00431_Suplementary Material**

**Supplementary Table 1** Colorectal cancer screening economic evaluations.

| **Economic evaluations included** |
| --- |
| Tsuji I, Fukao A, Shoji T, Kuwajima I, Sugawara N, Hisamichi S. Cost-effectiveness analysis of screening for colorectal cancer in Japan. Tohoku J Exp Med. 1991 Aug;164(4):269-78. |
| Wagner JL, Herdman RC, Wadhwa S. Cost effectiveness of colorectal cancer screening in the elderly. Ann Intern Med. 1991 Nov 15;115(10):807-17. |
| Shimbo T, Glick HA, Eisenberg JM. Cost-effectiveness analysis of strategies for colorectal cancer screening in Japan. Int J Technol Assess Health Care. 1994 Summer;10(3):359-75. |
| Lieberman DA. Cost-effectiveness model for colon cancer screening. Gastroenterology. 1995 Dec;109(6):1781-90. |
| Salkeld G, Young G, Irwig L, Haas M, Glasziou P. Cost-effectiveness analysis of screening by faecal occult blood testing for colorectal cancer in Australia. Aust N Z J Public Health. 1996 Apr;20(2):138-43. |
| Bolin, Korman, Stanton, Talley, Newstead, Donnelly, Hall, Ho, Lapsley. Positive cost effectiveness of early diagnosis of colorectal cancer. Colorectal Dis. 1999 Mar;1(2):113-22. |
| Sonnenberg A, Delcò F, Bauerfeind P. Is virtual colonoscopy a cost-effective option to screen for colorectal cancer? Am J Gastroenterol. 1999 Aug;94(8):2268-74. |
| Khandker RK, Dulski JD, Kilpatrick JB, Ellis RP, Mitchell JB, Baine WB. A decision model and cost-effectiveness analysis of colorectal cancer screening and surveillance guidelines for average-risk adults. Int J Technol Assess Health Care. 2000 Summer;16(3):799-810. |
| Sonnenberg A, Delcò F, Inadomi JM. Cost-effectiveness of colonoscopy in screening for colorectal cancer. Ann Intern Med. 2000 Oct 17;133(8):573-84. |
| Vijan S, Hwang EW, Hofer TP, Hayward RA. Which colon cancer screening test? A comparison of costs, effectiveness, and compliance. Am J Med. 2001 Dec 1;111(8):593-601. |
| Sonnenberg A, Delcò F. Cost-effectiveness of a single colonoscopy in screening for colorectal cancer. Arch Intern Med. 2002 Jan 28;162(2):163-8. |
| van Ballegooijen M, Habbema JDF, Boer R, Zauber AG, Brown ML. A Comparison of the Cost-Effectiveness of Fecal Occult Blood Tests with Different Test Characteristics in the Context of Annual Screening in the Medicare Population (Internet). Rockville (MD): Agency for Healthcare Research and Quality (US); 2003 Aug 9. |
| O'Leary BA, Olynyk JK, Neville AM, Platell CF. Cost-effectiveness of colorectal cancer screening: comparison of community-based flexible sigmoidoscopy with fecal occult blood testing and colonoscopy. J Gastroenterol Hepatol. 2004 Jan;19(1):38-47 |
| Berchi C, Bouvier V, Réaud JM, Launoy G. Cost-effectiveness analysis of two strategies for mass screening for colorectal cancer in France. Health Econ. 2004 Mar;13(3):227-38. |
| Lejeune C, Arveux P, Dancourt V, Béjean S, Bonithon-Kopp C, Faivre J. Cost-effectiveness analysis of fecal occult blood screening for colorectal cancer. Int J Technol Assess Health Care. 2004 Fall;20(4):434-9. |
| Wong SS, Leong AP, Leong TY. Cost-effectiveness analysis of colorectal cancer screening strategies in Singapore: a dynamic decision analytic approach. Stud Health Technol Inform. 2004;107(Pt 1):104-10. |
| Ladabaum U, Song K, Fendrick AM. Colorectal neoplasia screening with virtual colonoscopy: when, at what cost, and with what national impact? Clin Gastroenterol Hepatol. 2004 Jul;2(7):554-63. |
| Song K, Fendrick AM, Ladabaum U. Fecal DNA testing compared with conventional colorectal cancer screening methods: a decision analysis. Gastroenterology. 2004 May;126(5):1270-9. |
| Wu GH, Wang YM, Yen AM, Wong JM, Lai HC, Warwick J, Chen TH. Cost-effectiveness analysis of colorectal cancer screening with stool DNA testing in intermediate-incidence countries. BMC Cancer. 2006 May 24;6:136. |
| Maciosek MV, Solberg LI, Coffield AB, Edwards NM, Goodman MJ. Colorectal cancer screening: health impact and cost effectiveness. Am J Prev Med. 2006 Jul;31(1):80-9. |
| Tappenden P, Chilcott J, Eggington S, Patnick J, Sakai H, Karnon J. Option appraisal of population-based colorectal cancer screening programmes in England. Gut. 2007 May;56(5):677-84. |
| Hassan C, Zullo A, Laghi A, Reitano I, Taggi F, Cerro P, Iafrate F, Giustini M, Winn S, Morini S. Colon cancer prevention in Italy: cost-effectiveness analysis with CT colonography and endoscopy. Dig Liver Dis. 2007 Mar;39(3):242-50. |
| Pickhardt PJ, Hassan C, Laghi A, Zullo A, Kim DH, Morini S. Cost-effectiveness of colorectal cancer screening with computed tomography colonography: the impact of not reporting diminutive lesions. Cancer. 2007 Jun 1;109(11):2213-21. |
| Vijan S, Hwang I, Inadomi J, Wong RK, Choi JR, Napierkowski J, Koff JM, Pickhardt PJ. The cost-effectiveness of CT colonography in screening for colorectal neoplasia. Am J Gastroenterol. 2007 Feb;102(2):380-90. |
| Zauber AG, Lansdorp-Vogelaar I, Wilschut J, Knudsen AB, van Ballegooijen M, Kuntz KM. Cost-Effectiveness of DNA Stool Testing to Screen for Colorectal Cancer (Internet). Rockville (MD): Agency for Healthcare Research and Quality (US); 2007 Dec 20. |
| Tsoi KK, Ng SS, Leung MC, Sung JJ. Cost-effectiveness analysis on screening for colorectal neoplasm and management of colorectal cancer in Asia. Aliment Pharmacol Ther. 2008 Aug 1;28(3):353-63. |
| Hassan C, Zullo A, Winn S, Morini S. Cost-effectiveness of capsule endoscopy in screening for colorectal cancer. Endoscopy. 2008 May;40(5):414-21. |
| Parekh M, Fendrick AM, Ladabaum U. As tests evolve and costs of cancer care rise: reappraising stool-based screening for colorectal neoplasia. Aliment Pharmacol Ther. 2008 Apr;27(8):697-712. |
| Regge D, Hassan C, Pickhardt PJ, Laghi A, Zullo A, Kim DH, Iafrate F, Morini S. Impact of computer-aided detection on the cost-effectiveness of CT colonography. Radiology. 2009 Feb;250(2):488-97. |
| Tafazzoli A, Roberts S, Klein R, Ness R, Dittus R. Probabilistic cost-effectiveness comparison of screening strategies for colorectal cancer. ACM Trans Model Comput Simul. 2009 mar;19(2):1-29. |
| Heitman SJ, Hilsden RJ, Au F, Dowden S, Manns BJ. Colorectal cancer screening for average-risk North Americans: an economic evaluation. PLoS Med. 2010 Nov 23;7(11):e1000370. |
| Telford JJ, Levy AR, Sambrook JC, Zou D, Enns RA. The cost-effectiveness of screening for colorectal cancer. CMAJ. 2010 Sep 7;182(12):1307-13. |
| Heresbach D, Chauvin P, Grolier J, Josselin JM. Cost-effectiveness of colorectal cancer screening with computed tomography colonography or fecal blood tests. Eur J Gastroenterol Hepatol. 2010 Nov;22(11):1372-9. |
| Lejeune C, Dancourt V, Arveux P, Bonithon-Kopp C, Faivre J. Cost-effectiveness of screening for colorectal cancer in France using a guaiac test versus an immunochemical test. Int J Technol Assess Health Care. 2010 Jan;26(1):40-7. |
| Lee D, Muston D, Sweet A, Cunningham C, Slater A, Lock K. Cost effectiveness of CT colonography for UK NHS colorectal cancer screening of asymptomatic adults aged 60-69 years. Appl Health Econ Health Policy. 2010;8(3):141-54. |
| Knudsen AB, Lansdorp-Vogelaar I, Rutter CM, Savarino JE, van Ballegooijen M, Kuntz KM, Zauber AG. Cost-effectiveness of computed tomographic colonography screening for colorectal cancer in the medicare population. J Natl Cancer Inst. 2010 Aug 18;102(16):1238-52. |
| Lansdorp-Vogelaar I, Kuntz KM, Knudsen AB, Wilschut JA, Zauber AG, van Ballegooijen M. Stool DNA testing to screen for colorectal cancer in the Medicare population: a cost-effectiveness analysis. Ann Intern Med. 2010 Sep 21;153(6):368-77. |
| Hassan C, Benamouzig R, Spada C, Ponchon T, Zullo A, Saurin JC, Costamagna G. Cost effectiveness and projected national impact of colorectal cancer screening in France. Endoscopy. 2011 Sep;43(9):780-93. |
| van Rossum LG, van Rijn AF, Verbeek AL, van Oijen MG, Laheij RJ, Fockens P, Jansen JB, Adang EM, Dekker E. Colorectal cancer screening comparing no screening, immunochemical and guaiac fecal occult blood tests: a cost-effectiveness analysis. Int J Cancer. 2011 Apr 15;128(8):1908-17. |
| Vanness DJ, Knudsen AB, Lansdorp-Vogelaar I, Rutter CM, Gareen IF, Herman BA, Kuntz KM, Zauber AG, van Ballegooijen M, Feuer EJ, Chen MH, Johnson CD. Comparative economic evaluation of data from the ACRIN National CT Colonography Trial with three cancer intervention and surveillance modeling network microsimulations. Radiology. 2011 Nov;261(2):487-98. |
| Tran B, Keating CL, Ananda SS, Kosmider S, Jones I, Croxford M, Field KM, Carter RC, Gibbs P. Preliminary analysis of the cost-effectiveness of the National Bowel Cancer Screening Program: demonstrating the potential value of comprehensive real world data. Intern Med J. 2012 Jul;42(7):794-800. |
| Wang ZH, Gao QY, Fang JY. Repeat colonoscopy every 10 years or single colonoscopy for colorectal neoplasm screening in average-risk Chinese: a cost-effectiveness analysis. Asian Pac J Cancer Prev. 2012;13(5):1761-6. |
| Pinzon Florez CE, Rosselli D, Gamboa Garay OA. Análisis de Costo-Efectividad de las Estrategias de Tamización de Cáncer Colorrectal en Colombia. Value Health Reg Issues. 2012 Dec;1(2):190-200. |
| Chauvin P, Josselin JM, Heresbach D. Incremental net benefit and acceptability of alternative health policies: a case study of mass screening for colorectal cancer. Eur J Health Econ. 2012 Jun;13(3):237-50. |
| Lucidarme O, Cadi M, Berger G, Taieb J, Poynard T, Grenier P, Beresniak A. Cost-effectiveness modeling of colorectal cancer: computed tomography colonography vs colonoscopy or fecal occult blood tests. Eur J Radiol. 2012 Jul;81(7):1413-9. |
| Barouni M, Larizadeh MH, Sabermahani A, Ghaderi H. Markov's modeling for screening strategies for colorectal cancer. Asian Pac J Cancer Prev. 2012;13(10):5125-9. |
| Sharp L, Tilson L, Whyte S, O'Ceilleachair A, Walsh C, Usher C, Tappenden P, Chilcott J, Staines A, Barry M, Comber H. Cost-effectiveness of population-based screening for colorectal cancer: a comparison of guaiac-based faecal occult blood testing, faecal immunochemical testing and flexible sigmoidoscopy. Br J Cancer. 2012 Feb 28;106(5):805-16. |
| Dan YY, Chuah BY, Koh DC, Yeoh KG. Screening based on risk for colorectal cancer is the most cost-effective approach. Clin Gastroenterol Hepatol. 2012 Mar;10(3):266-71.e1-6. |
| Dinh T, Ladabaum U, Alperin P, Caldwell C, Smith R, Levin TR. Health benefits and cost-effectiveness of a hybrid screening strategy for colorectal cancer. Clin Gastroenterol Hepatol. 2013 Sep;11(9):1158-66. |
| Ladabaum U, Allen J, Wandell M, Ramsey S. Colorectal cancer screening with blood-based biomarkers: cost-effectiveness of methylated septin 9 DNA versus current strategies. Cancer Epidemiol Biomarkers Prev. 2013 Sep;22(9):1567-76 |
| Sharaf RN, Ladabaum U. Comparative effectiveness and cost-effectiveness of screening colonoscopy vs. sigmoidoscopy and alternative strategies. Am J Gastroenterol. 2013 Jan;108(1):120-32. |
| Ladabaum U, Alvarez-Osorio L, Rösch T, Brueggenjuergen B. Cost-effectiveness of colorectal cancer screening in Germany: current endoscopic and fecal testing strategies versus plasma methylated Septin 9 DNA. Endosc Int Open. 2014 Jun;2(2):E96-E104. |
| Lejeune C, Le Gleut K, Cottet V, Galimard C, Durand G, Dancourt V, Faivre J. The cost-effectiveness of immunochemical tests for colorectal cancer screening. Dig Liver Dis. 2014 Jan;46(1):76-81. |
| Wong CK, Lam CL, Wan YF, Fong DY. Cost-effectiveness simulation and analysis of colorectal cancer screening in Hong Kong Chinese population: comparison amongst colonoscopy, guaiac and immunologic fecal occult blood testing. BMC Cancer. 2015 Oct 15;15:705. |
| Hassan C, Gralnek IM. Cost-effectiveness of "full spectrum endoscopy" colonoscopy for colorectal cancer screening. Dig Liver Dis. 2015 May;47(5):390-4. |
| Espinola N, Maceira D, Palacios A. Costo-efectivided de las pruebas de tamizaje del cáncer colorectal en la Argentina (Cost-effectiveness of screening for colorectal cancer in Argentina.). Acta Gastroenterol Latinoam. 2016 Mar;46(1):8-17. Spanish. |
| Pil L, Fobelets M, Putman K, Trybou J, Annemans L. Cost-effectiveness and budget impact analysis of a population-based screening program for colorectal cancer. Eur J Intern Med. 2016 Jul;32:72-8. |
| Greuter MJ, Berkhof J, Fijneman RJ, Demirel E, Lew JB, Meijer GA, Stoker J, Coupé VM. The potential of imaging techniques as a screening tool for colorectal cancer: a cost-effectiveness analysis. Br J Radiol. 2016 Jul;89(1063):20150910. |
| Sekiguchi M, Igarashi A, Matsuda T, Matsumoto M, Sakamoto T, Nakajima T, Kakugawa Y, Yamamoto S, Saito H, Saito Y. Optimal use of colonoscopy and fecal immunochemical test for population-based colorectal cancer screening: a cost-effectiveness analysis using Japanese data. Jpn J Clin Oncol. 2016 Feb;46(2):116-25. |
| Berger BM, Schroy PC 3rd, Dinh TA. Screening for Colorectal Cancer Using a Multitarget Stool DNA Test: Modeling the Effect of the Intertest Interval on Clinical Effectiveness. Clin Colorectal Cancer. 2016 Sep;15(3):e65-74. |
| Ladabaum U, Mannalithara A. Comparative Effectiveness and Cost Effectiveness of a Multitarget Stool DNA Test to Screen for Colorectal Neoplasia. Gastroenterology. 2016 Sep;151(3):427-439.e6. |
| Hasdeu S, Lamfre L, Torales S, Caporale J, Sánchez-Viamonte J, Hutter F, MacMullen M. (Costo-efectividad del rastreo de cáncer colorrectal en provincias argentinas selecionadas). Rev. argent. salud publica; 8(31): 13-18, jun. 2017. Spanish |
| Lew JB, St John DJB, Xu XM, Greuter MJE, Caruana M, Cenin DR, He E, Saville M, Grogan P, Coupé VMH, Canfell K. Long-term evaluation of benefits, harms, and cost-effectiveness of the National Bowel Cancer Screening Program in Australia: a modelling study. Lancet Public Health. 2017 Jul;2(7):e331-e340. |
| Goede SL, Rabeneck L, van Ballegooijen M, Zauber AG, Paszat LF, Hoch JS, Yong JH, Kroep S, Tinmouth J, Lansdorp-Vogelaar I. Harms, benefits and costs of fecal immunochemical testing versus guaiac fecal occult blood testing for colorectal cancer screening. PLoS One. 2017 Mar 15;12(3):e0172864. |
| Greuter MJE, de Klerk CM, Meijer GA, Dekker E, Coupé VMH. Screening for Colorectal Cancer with Fecal Immunochemical Testing With and Without Postpolypectomy Surveillance Colonoscopy: A Cost-Effectiveness Analysis. Ann Intern Med. 2017 Oct 17;167(8):544-554. |
| Murphy J, Halloran S, Gray A. Cost-effectiveness of the faecal immunochemical test at a range of positivity thresholds compared with the guaiac faecal occult blood test in the NHS Bowel Cancer Screening Programme in England. BMJ Open. 2017 Oct 27;7(10):e017186. |
| Aronsson M, Carlsson P, Levin LÅ, Hager J, Hultcrantz R. Cost-effectiveness of high-sensitivity faecal immunochemical test and colonoscopy screening for colorectal cancer. Br J Surg. 2017 Jul;104(8):1078-1086. |
| Barzi A, Lenz HJ, Quinn DI, Sadeghi S. Comparative effectiveness of screening strategies for colorectal cancer. Cancer. 2017 May 1;123(9):1516-1527. |
| Lew JB, St John DJB, Macrae FA, Emery JD, Ee HC, Jenkins MA, He E, Grogan P, Caruana M, Sarfati D, Greuter MJE, Coupé VMH, Canfell K. Evaluation of the benefits, harms and cost-effectiveness of potential alternatives to iFOBT testing for colorectal cancer screening in Australia. Int J Cancer. 2018 Jul 15;143(2):269-282. |
| Lansdorp-Vogelaar I, Goede SL, Bosch LJW, Melotte V, Carvalho B, van Engeland M, Meijer GA, de Koning HJ, van Ballegooijen M. Cost-effectiveness of High-performance Biomarker Tests vs Fecal Immunochemical Test for Noninvasive Colorectal Cancer Screening. Clin Gastroenterol Hepatol. 2018 Apr;16(4):504-512.e11. |
| van der Meulen MP, Lansdorp-Vogelaar I, Goede SL, Kuipers EJ, Dekker E, Stoker J, van Ballegooijen M. Colorectal Cancer: Cost-effectiveness of Colonoscopy versus CT Colonography Screening with Participation Rates and Costs. Radiology. 2018 Jun;287(3):901-911. |
| Senore C, Hassan C, Regge D, Pagano E, Iussich G, Correale L, Segnan N. Cost-effectiveness of colorectal cancer screening programmes using sigmoidoscopy and immunochemical faecal occult blood test. J Med Screen. 2019 Jun;26(2):76-83. |
| Arrospide A, Idigoras I, Mar J, de Koning H, van der Meulen M, Soto-Gordoa M, Martinez-Llorente JM, Portillo I, Arana-Arri E, Ibarrondo O, Lansdorp-Vogelaar I. Cost-effectiveness and budget impact analyses of a colorectal cancer screening programme in a high adenoma prevalence scenario using MISCAN-Colon microsimulation model. BMC Cancer. 2018 Apr 25;18(1):464. |
| Melnitchouk N, Soeteman DI, Davids JS, Fields A, Cohen J, Noubary F, Lukashenko A, Kolesnik OO, Freund KM. Cost-effectiveness of colorectal cancer screening in Ukraine. Cost Eff Resour Alloc. 2018 Jun 7;16:20. |
| Jahn B, Sroczynski G, Bundo M, Mühlberger N, Puntscher S, Todorovic J, Rochau U, Oberaigner W, Koffijberg H, Fischer T, Schiller-Fruehwirth I, Öfner D, Renner F, Jonas M, Hackl M, Ferlitsch M, Siebert U; Austrian Colorectal Cancer Screening Model Group. Effectiveness, benefit harm and cost effectiveness of colorectal cancer screening in Austria. BMC Gastroenterol. 2019 Dec 5;19(1):209. |
| Areia M, Fuccio L, Hassan C, Dekker E, Dias-Pereira A, Dinis-Ribeiro M. Cost-utility analysis of colonoscopy or faecal immunochemical test for population-based organised colorectal cancer screening. United European Gastroenterol J. 2019 Feb;7(1):105-113. |
| Phisalprapa P, Supakankunti S, Chaiyakunapruk N. Cost-effectiveness and budget impact analyses of colorectal cancer screenings in a low- and middle-income country: example from Thailand. J Med Econ. 2019 Dec;22(12):1351-1361. |
| Naber SK, Knudsen AB, Zauber AG, Rutter CM, Fischer SE, Pabiniak CJ, Soto B, Kuntz KM, Lansdorp-Vogelaar I. Cost-effectiveness of a multitarget stool DNA test for colorectal cancer screening of Medicare beneficiaries. PLoS One. 2019 Sep 4;14(9):e0220234. |
| Barré S, Leleu H, Benamouzig R, Saurin JC, Vimont A, Taleb S, De Bels F. Cost-effectiveness analysis of alternative colon cancer screening strategies in the context of the French national screening program. Therap Adv Gastroenterol. 2020 Sep 20;13: 1756284820953364. |

**Supplementary Table 2** Main characteristics of colorectal cancer screening economic evaluations.

| **Authors** | **Country** | **Study** | **Model type** | **Perspective** | **Time horizon (years)** | **Screening age range (years)** | **CRC screening strategies** | **‘No screening’ comparator** |
| --- | --- | --- | --- | --- | --- | --- | --- | --- |
| Tsuji et al. | Japan | CEA | NR | Third-party | 40 | 40‒79 | FIT annual; DCBE, FS and COL: NR | Yes |
| Wagner et al. | USA | CEA | NR | Third-party | 20 | 65‒85 | FOBT annual; FOBT annual + FS 3-yearly; FOBT annual + FS 5-yearly; FOBT annual + FS once at age 65 | Yes |
| Shimbo et al. | Japan | CEA | Markov | Third-party | 35 | 40‒60 | Biochemical FOBT annual; FIT annual; FIT biennial | Yes |
| Lieberman | USA | CEA | NR | Third-party | 10 | 55‒65 | gFOBT annual; FS 5-yearly; gFOBT annual + FS biennial; DCBE 5-yearly; COL only once | Yes |
| Salkeld et al. | Australia | CEA | NR | Third-party | NR | 50‒80 | gFOBT annual | Yes |
| Bolin et al. | Australia | CEA | Decision tree | Third-party | 35 | 50‒85 | gFOBT annual; gFOBT 3-yearly; FS 3-yearly; FS 5-yearly; gFOBT annual + FS 3-yearly; gFOBT annual + FS 5-yearly; COL: once at age 50; COL 5-yearly; COL 10-yearly; DCBE 3-yearly; DCBE 5-yearly | Yes |
| Sonnenberg et al. | USA | CEA | Markov | Third-party | Lifetime | 50 ‒ X | CT 10-yearly; COL 10-yearly | Yes |
| Khandker et al. | USA | CEA | NR | Third-party | 20 | 50‒85 | FOBT annual; FS 3-yearly; FS 5-yearly; FOBT annual + FS 5-yearly; FOBT annual + FS 3-yearly; DCBE 5-yearly; COL 5-yearly; COL 10-yearly | Yes |
| Sonnenberg et al. | USA | CEA | Markov | Third-party | Lifetime | 50 ‒ X | FOBT annual; FS 5-yearly; COL 10-yearly | Yes |
| Vijan et al. | USA | CEA | Markov | Third-party | Lifetime | 50‒80 | FOBT: NR, FS: NR, FOBT+FS: NR, COL once at age 55, once at age 60, twice at 50 and 60 | Yes |
| Sonnenberg et al. | USA | CEA | Markov | Third-party | Lifetime | 65‒75 | COL 10-yearly; COL once at age 65 | Yes |
| van Ballegooijen et al. | USA | CEA | Microsimulation | Third-party | Lifetime | 65‒79 | gFOBT annual; FIT annual | Yes |
| O'Leary et al. | Australia | CEA | Markov | Third-party | 10 | 55‒64 | gFOBT annual; gFOBT biennial; FS 10-yearly; COL 10-yearly | Yes |
| Berchi et al. | France | CEA | Markov | Third-party | 20 | 50‒74 | FIT biennial; gFOBT biennial | No (gFOBT comparator) |
| Lejeune et al. | France | CEA | Markov | Third-party | 20 | 50‒74 | gFOBT biennial | Yes |
| Wong et al. | Singapore | CEA | Markov | NR | 50 | 50‒70 | gFOBT annual; FIT annual; FS 3-yearly; DCBE 5-yearly; COL 10-yearly | Yes |
| Ladabaum et al. | USA | CEA | Markov | Third-party | Lifetime | 50‒80 | Virtual COL 10-yearly; COL 10-yearly | Yes |
| Song et al. | USA | CEA | Markov | Third-party | Lifetime | 50‒80 | COL 10-yearly; FOBT annual; FS 5-yearly; Stool DNA 5-yearly | Yes |
| Wu et al. | Taiwan | CEA | Markov | Third-party | 25 | 50‒75 | FOBT annual; Stool DNA 3-yearly; Stool DNA 5-yearly; Stool DNA 10-yearly; FS 5-yearly; COL 10-yearly | Yes |
| Maciosek et al. | USA | CEA | NR | Society | Lifetime | 50‒80 | FOBT annual; FS 5-yearly; COL 10-yearly | Yes |
| Tappenden et al. | England | CEA | Markov | NR | Lifetime | 50‒69 | FOBT biennial; FS once at age 55; FS once at age 60; FS once at age 60 + FOBT biennial | Yes |
| Hassan et al. | Italy | CEA | Markov | NR | 50 | 50‒80 | FS 10-yearly; CT 10-yearly; COL 10-yearly | Yes |
| Pickhardt et al. | USA | CEA | Markov | NR | 30 | 50‒80 | FS 10-yearly; CT 10-yearly; CT 10-yearly (less sensitivity); Optical COL 10-yearly | Yes |
| Vijan et al. | USA | CEA | Markov | Third-party/Society | Lifetime | 50‒80 | FOBT annual; FS 5-yearly; FOBT annual + FS 5-yearly; CT 5-yearly; CT 10-yearly; COL 10-yearly | Yes |
| Zauber et al. | USA | CEA | Microsimulation | Third-party/Society | Lifetime | 65‒80 | Stool DNA 3-yearly; Stool DNA 5-yearly; gFOBT annual; FIT annual; FS 5-yearly; FIT annual + FS 5-yearly; gFOBT annual + FS 5-yearly; COL 10-yearly | Yes |
| Tsoi et al. | Asia | CEA | Markov | NR | Lifetime | 50‒80 | gFOBT annual; FS 5-yearly; COL 10-yearly | Yes |
| Hassan et al. | USA | CEA | Markov | Society | Lifetime | 50‒80 | Capsule Endoscopy 10-yearly; COL 10-yearly | Yes |
| Parekh et al. | USA | CEA | Markov | Third-party | Lifetime | 50‒80 | gFOBT annual; FIT annual; Stool DNA 3-yearly | Yes |
| Regge et al. | USA | CEA | Markov | Society | Lifetime | 50‒80 | CT com CAD (Computer-aided diagnosis) 10-yearly; CT 10-yearly; Optical COL 10-yearly; FS 10-yearly | Yes |
| Tafazzoli et al. | USA | CUA | Microsimulation | NR | Lifetime | 50‒80 | FOBT annual; FS 5-yearly; COL 10-yearly; COL virtual 5-yearly COL virtual 10-yearly; Stool DNA 3-yearly; Stool DNA 5-yearly; DCBE 5-yearly | Yes |
| Heitman et al. | Canada | CUA | Markov | Third-party | Lifetime | 50‒75 | FIT annual; gFOBT annual; Stool DNA 3-yearly; CT 5-yearly; FS 5-yearly; COL 10-yearly | Yes |
| Telford et al. | Canada | CUA | Markov | Third-party | Lifetime | 50‒75 | gFOBT annual; gFOBT biennial; FIT annual; FS 5-yearly; gFOBT annual + FS 5-yearly; Stool DNA 3-yearly; DCBE 5-yearly; CT 5-yearly; COL 10-yearly | Yes |
| Heresbach et al. | France | CEA | Markov | Third-party | 30 | 50‒74 | FIT biennial; gFOBT biennial; CT 10-yearly | Yes |
| Lejeune et al. | France | CEA | Markov | Third-party | 20 (or until death) | 50‒74 | gFOBT biennial; FIT biennial | Yes |
| Lee et al. | United Kingdom | CUA | Markov | Third-party | Lifetime | 60‒69 | gFOBT biennial; CT 10-yearly; FS 10-yearly; Optical COL 10-yearly | No (gFOBT comparator) |
| Knudsen et al. | USA | CEA | Microsimulation | Third-party | Lifetime | 65‒80 | CT 5-yearly; FIT annual; gFOBT annual; FS 5-yearly; FS 5-yearly; FIT annual + FS 5-yearly; COL 10-yearly | Yes |
| Lansdorp-Vogelaar et al. | USA | CEA | Microsimulation | Third-party | Lifetime | 65‒80 | FIT annual; gFOBT annual; FS 5-yearly; gFOBT annual + FS 5-yearly; FIT annual + FS 5-yearly; Stool DNA 3-yearly; Stool DNA 5-yearly; COL 10-yearly | Yes |
| Hassan et al. | France | CEA | Markov | Third-party | Lifetime | 50‒75 | gFOBT annual; gFOBT biennial; FIT annual; FIT biennial; FS 5-yearly; FS 10-yearly; Capsule Endoscopy 5-yearly; Capsule Endoscopy 10-yearly; COL 10-yearly | Yes |
| van Rossum et al. | Netherlands | CEA | Markov | Third-party | 10 | 50‒75 | gFOBT annual; FIT annual | Yes |
| Vanness et al. | USA | CEA | Microsimulation | Third-party | Lifetime | 50‒80 | gFOBT annual + FS 5-yearly; FIT annual + FS 5-yearly; CT 5-yearly; CT 10-yearly; FS 5-yearly; COL 10-yearly | Yes |
| Tran et al. | Australia | CEA | NR | Third-party | Lifetime | 50‒74 | FOBT biennial | Yes |
| Wang et al. | China | CEA | Markov | Third-party | NR | 50‒80 | COL only once; COL 10-yearly | Yes |
| Pinzon Florez et al. | Colombia | CEA | Markov | Third-party | Lifetime | 50‒70 | gFOBT annual; gFOBT biennial; FIT annual; FIT biennial; FS 5-yearly; FIT annual + FS 5-yearly; COL 10-yearly | Yes |
| Chauvin et al. | France | CEA | Markov | Third-party | 30 | 50‒80 | gFOBT biennial; FIT biennial; CT 5-yearly; CT 10-yearly | No (gFOBT comparator) |
| Lucidarme et al. | France | CEA | Markov | Third-party | 10 | 50‒74 | FOBT biennial; CT 10-yearly; Optical COL 10-yearly | Yes |
| Barouni et al. | Iran | CEA | Markov | Third-party | Lifetime | 50‒75 | gFOBT annual; gFOBT biennial; FIT annual; FS 5-yearly; gFOBT annual + FS 5-yearly; CT 5-yearly; DCBE 5-yearly; Stool DNA 3-yearly; COL 10-yearly | Yes |
| Sharp et al. | Ireland | CUA | Markov | Third-party | 100 | 55‒74 | gFOBT biennial; FIT biennial; FS once at age 60; FS once at age 55 | Yes |
| Dan et al. | Singapore | CUA | Markov | Society | Lifetime | 50‒75 | FIT: annual; FS once at age 60; FS 5-yearly; FIT annual + FS 5-yearly; COL once at age 60; COL 10-yearly; DCBE 5-yearly; Stool DNA 5-yearly; CT 5-yearly; FIT annual (50-60) + COL 10-yearly (60-72) | Yes |
| Dinh et al. | USA | CUA | Microsimulation | Society | Lifetime | 50‒75 | FIT annual; COL 10-yearly; FS 5-yearly; FIT annual + FS 10-yearly, FIT annual (from 50) + COL once at age 66 | Yes |
| Ladabaum et al. | USA | CUA | Markov | Third-party | Lifetime | 50‒80 | gFOBT annual; FIT annual; FS 5-yearly; Plasma DNA biennial; COL 10-yearly | Yes |
| Sharaf et al. | USA | CUA | Markov | Third-party | Lifetime | 50‒80 | gFOBT annual; FIT annual; FS: once at age 60; FS 5-yearly; FIT + RS: 3-yearly; COL 10-yearly | Yes |
| Ladabaum et al. | Germany | CUA | Markov | Third-party | Lifetime | 50‒75 | FIT annual (50‒54) + biennial (55‒75); gFOBT annual (50‒54) + biennial (55‒75); gFOBT annual; gFOBT biennial; FIT annual; FIT biennial; Plasma DNA biennial; COL 10-yearly (at 55 and 65 or 60 and 70 years); COL 10-yearly | Yes |
| Lejeune et al. | France | CEA | Markov | Third-party | 20 | 50‒74 | gFOBT biennial; FIT biennial | No (gFOBT comparator) |
| Wong et al. | Hong Kong | CUA | Markov | Third-party | 25 | 50‒75 | gFOBT annual; gFOBT biennial; FIT annual; FIT biennial; COL 10-yearly | Yes |
| Hassan et al. | USA | CEA | Markov | Society | Lifetime | 50‒100 | END total (FUSE) 10-yearly; COL 10-yearly | Yes |
| Espinola et al. | Argentina | CUA | Markov | Third-party | 50 | 50‒74 | FIT annual; COL 10-yearly | Yes |
| Pil et al. | Belgium | CUA | Markov | Society | 20 | 50‒74 | FIT biennial | Yes |
| Greuter et al. | Netherlands | CEA | Markov | Third-party | Lifetime | 50‒80 | FIT biennial; CT 5-yearly; CT 10-yearly; MRI 5-yearly; MRI 10-yearly; COL 10-yearly | Yes |
| Sekiguchi et al. | Japan | CUA | Markov | Third-party | Lifetime | 40 ‒ X | FIT annual; COL 10-yearly; FIT annual (40‒49) + COL (once at age 50) + FIT 5-yearly (if COL negative, 51‒X) | Yes |
| Berger et al. | USA | CUA | Simulation (Archimedes model) | Society | 30 | 50‒85 | Stool DNA annual; Stool DNA 3-yearly; Stool DNA 5-yearly; COL 10-yearly | Yes |
| Ladabaum et al. | USA | CUA | Markov | Third-party | Lifetime | 50‒80 | FIT annual; FIT biennial; Stool DNA 3-yearly; COL 10-yearly | Yes |
| Hasdeu et al. | Argentina | CEA | Markov | Third-party | Lifetime | 50‒74 | FIT annual | Yes |
| Lew et al. | Australia | CEA | Microsimulation | Third-party | Lifetime | 50‒74 | FIT biennial | Yes |
| Goede et al. | Canada | CUA | Microsimulation | Third-party | Lifetime | 40‒85 | gFOBT annual; gFOBT 1.5-yearly; gFOBT biennial; gFOBT 3-yearly; FIT annual; FIT 1.5-yearly; FIT biennial; FIT 3-yearly | Yes |
| Greuter et al. | Netherlands | CEA | Microsimulation | Third-party | Lifetime | 55‒75 | FIT biennial; FIT + COL 5-yearly; FIT + COL 10-yearly | Yes |
| Murphy et al. | United Kingdom | CUA | Markov | Third-party | 100 | 60‒74 | gFOBT annual; FIT annual | No (gFOBT comparator) |
| Aronsson et al. | Sweden | CUA | Markov | Third-party | Lifetime | 60‒80 | FIT biennial; FIT only twice; COL 10-yearly; COL only once | Yes |
| Barzi et al. | USA | CEA | Markov | Society | 35 (or until death) | 50‒75 | gFOBT annual; gFOBT biennial; FIT annual; FIT biennial; Stool DNA annual; Stool DNA biennial; gFOBT annual + FS 5-yearly; FIT annual + FS 5-yearly; gFOBT biennial + FS 5-yearly; FIT biennial + FS 5-yearly; FS 5-yearly; CT 10-yearly; COL 10-yearly | Yes |
| Lew et al | Australia | CEA | Microsimulation | Third-party | Lifetime | 50‒74 | FIT annual; FIT biennial; FS 10-yearly; FS once at age 60; CT 10-yearly; Stool DNA 5-yearly; Plasma DNA biennial; FS once at age 55 + FIT biennial (60‒74); FIT biennial + FS 10-yearly (54, 64 and 74); FIT biennial + FS once at age 50; COL once at age 50 + FIT biennial (52‒74); FIT biennial (50‒74) + Plasma DNA; COL 10-yearly | Yes |
| Lansdorp-Vogelaar et al. | Netherlands | CEA | Microsimulation | Society | Lifetime | 55‒75 | FIT biennial; Stool DNA biennial | Yes |
| van der Meulen et al. | Netherlands | CUA | Microsimulation | Third-party | Lifetime | 40‒65 | CT 3-yearly; CT 5-yearly; COL 10-yearly | Yes |
| Senore et al. | Italy | CEA | Markov | Third-party | 12 | 58‒70 | FIT biennial; FS once at age 58; FS once at age 58 + FIT (for nonadherents) | Yes |
| Arrospide et al. | Basque Country | CUA | Microsimulation | Third-party | Lifetime | 50‒69 | FIT biennial | Yes |
| Melnitchouk et al. | Ukraine | CUA | Markov | Third-party | Lifetime | 50‒75 | FOBT annual; FOBT annual + FS 5-yearly; COL 10-yearly | Yes |
| Jahn et al. | Austria | CEA | Markov | Third-party | Lifetime | 40‒75 | FIT annual; gFOBT annual; COL 10-yearly | Yes |
| Areia et al. | Portugal | CUA | Markov | Society | NR | 50‒74 | FIT biennial; COL 10-yearly | Yes |
| Phisalprapa et al. | Thailand | CUA | Markov | Society | Lifetime | 50‒75 | FIT annual; COL 10-yearly | Yes |
| Naber et al. | USA | CEA | Microsimulation | Third-party | Lifetime | 65‒75 | gFOBT annual; FIT annual; Stool DNA 3-yearly; FS 5-yearly; gFOBT annual + FS 10-yearly; FIT annual + FS 10-yearly; COL 10-yearly | Yes |
| Barré et al. | France | CUA | Microsimulation | Society | 80 | 50‒74 | gFOBT biennial; FIT biennial; Stool DNA biennial; Biomarkers biennial; CT 10-yearly; Colon capsule 10-yearly; FS 10-yearly | Yes |

CEA, Cost-Effectiveness Analysis; COL, Colonoscopy; CT, Computed Tomography; CUA, Cost-Utility Analysis; DCBE, Double Contrast Barium Enema; FIT, Fecal Immunochemical Test; FOBT, Fecal Occult Blood Test; FS, Flexible Sigmoidoscopy; gFOBT, Guaiac Fecal Occult Blood Test; MRI, Magnetic Resonance Imaging; NR, Not Report.

**Supplementary Table 3** Cost-effectiveness, adherence rate in the baseline case and sensitivity analysis results.

| **Authors/Country** | **Cost-effective CRC screening strategy** | **ICER (International dollars – PPP*,* 2020)^a^** | **CET** | **Adherence rate (%)** | **Parameters most impacting ICER** |
| --- | --- | --- | --- | --- | --- |
| Hassan et al., USA | COL 10-yearly | 20,505.50/LYG | NR | 35–68 (FIT); 18–38 (COL) | Adherence rate; Test cost; Test specificity |
| Aronsson et al., Sweden | COL 10-yearly | Cost-saving | 10,000/QALY | 38 (COL); 50 (FIT) | Discount rate; Age screening started; Disease progression |
| Tafazzoli et al., USA | COL 10-yearly | NR | 50,000/QALY | 100 | Adherence rate |
| Tsuji et al., Japan | COL (frequency NR) | 376.20/LYG | NR | 68 | Complications cost COL; Test sensitivity (DCBE) |
| Sonnenberg et al., USA | COL 10-yearly | 16,585.11/LYG | NR | 29.1 (stool tests); 65 (blood tests); 25 (imaging) | Test cost (CT) |
| Maciosek et al., USA | COL 10-yearly | 13,574.22/LYG | NR | 100 | Adherence rate; Sensitivity test (COL); Life expectancy |
| Sonnenberg et al., USA | COL 10-yearly | 16,864.90/LYG | NR | 44 | Adherence rate |
| Ladabaum et al., USA | COL 10-yearly | 26,730.88/LYG | NR | 100 | Adherence rate; Cost ratio virtual COL and confirmatory COL |
| Melnitchouk et al., Ukraine | COL 10-yearly | Cost-saving | 11,700/QALY | 100 | Adherence rate (COL); Test cost (COL) |
| Sekiguchi et al., Japan | COL 10-yearly | Cost-saving | 5‒6 million/LYG | NR | Age screening started |
| Barzi et al., USA | COL 10-yearly | Cost-saving | 50,000/LYG | 100 | Adherence rate; Metastasis reduction |
| Wu et al., Taiwan | COL 10-yearly; FOBT annual | NR | 13,000/LYG | 100 | Adherence rate COL (secondary test); Test cost; Adenoma prevalence; Discount rate |
| Sonnenberg et al., USA | COL only once | 4,577.46/LYG | NR | 26.5 (COL); 60 (FIT) | Adherence rate; Test cost (COL); Cost and incidence of COL complications; CRC treatment cost |
| Wang et al., China | COL only once | 15.19/number of cases avoided | NR | 10000 | Adherence rate; CRC treatment cost |
| Vijan et al., USA | COL only once at age 60 | 203.99/LYG | NR | 34 (CT, MRI); 22 (COL); 63 (FIT) | Adherence rate; Test cost (COL); Disease progression; Discount rate |
| Lee et al., United Kingdom | CT 10-yearly | Cost-saving | 20,000‒30,000/QALY | NR | Adherence rate; Test cost |
| Hassan et al., Italy | CT 10-yearly | Cost-saving | NR | 60 | Adherence rate; Test cost; Screening frequency |
| Pickhardt et al., USA | CT 10-yearly | 5,531.98/LYG | NR | 65 | Adherence rate; Test cost |
| Berger et al., USA | Stool DNA 5-yearly | 8,804.20/QALY | 25,000/QALY | 100 | Test sensitivity |
| Hassan et al., USA | END FUSE 10-yearly | 17,284.60/LYG | 50,000/LYG | 40 | Adherence rate (COL); CRC incidence |
| Greuter et al., Netherlands | FOBT + COL 5-yearly/FOBT + COL 10-yearly | Cost-saving | 36,602/LYG | 100 | Test cost (COL); Complications cost COL |
| Lucidarme et al., France | FOBT biennial | NR | NR | 68 (first round) e 63 (subsequently rounds) | Adherence rate |
| Tran et al., Australia | FOBT biennial | 34,090.82/LYG | NR | 42 | Adherence rate |
| Tappenden et al., England | FOBT biennial | 3,506.69/LYG | NR | 100 | Test cost; Test specificity |
| Song et al., USA | FOBT annual | 10,237.36/LYG | 10,000‒50,000/LYG | 100 | Test cost; Test specificity |
| Vijan et al., USA | FOBT annual | 7,621.14/LYG | 50,000/LYG | 100 | Adherence rate; Test cost; Test specificity |
| Wong et al., Singapore | FOBT annual | 239.50/LYG | NR | 100 | Not evaluated |
| Wagner et al., USA | FOBT annual | 68,645.07/LYG | NR | 100 | Test sensitivity; Disease progression |
| Naber et al., USA | FOBT annual and COL 10-yearly | NR | 50,000-150,000/LYG | 100 | Test cost |
| Lejeune et al., France | gFOBT biennial | 6,691.41/LYG | NR | 100 | Adherence rate; Test cost |
| Heresbach et al., France | gFOBT biennial | 4,651.42/LYG | 20,000/LYG | 100 | Adherence rate; Test cost (TC) |
| Salkeld et al., Australia | gFOBT annual | 32,025.81/LYG | NR | NR | Confirmatory test cost (COL); Test specificity |
| Bolin et al., Australia | gFOBT annual | 37,000.41/LYG | NR | 60 | Discount rate |
| Lansdorp-Vogelaar, USA | gFOBT annual | 7,103.67/LYG | NR | 52.8–58.3 | Screening frequency |
| Lieberman, USA | gFOBT annual | 388,290.08/deaths avoided | NR | 55 | Adherence rate; Test sensitivity (gFOBT) |
| Zauber et al., USA | gFOBT annual | 761.11/LYG | NR | 5000 | Adherence rate |
| Tsoi et al., Asia | gFOBT annual | 7,673.98/LYG | NR | 40 | Test specificity (gFOBT); CRC treatment cost |
| Vanness et al., USA | gFOBT annual + FS 5-yearly | 3,805.54/LYG | 50,000/LYG | 100 | Adherence rate |
| Dan et al., Singapore | FIT annual (50–60) + COL 10-yearly (60‒72) | 28,214.76/QALY | 50,000/QALY | 100 | Adherence rate; Test cost (COL); CRC incidence; Test sensitivity; Test specificity; Risk of death by other causes |
| Lejeune et al., France | FIT biennial | 4,996.64/QALY | 20,000/QALY | 60 | Adherence rate; Test cost |
| Wong et al., Hong Kong | FIT biennial | 23,560.35/LYG, 3,534.41/QALY | 50,000/LYG or QALY | 60 | Test specificity (FIT) |
| Pinzon Florez et al., Colombia | FIT biennial | 12,330.85/LYG | NR | 75 (FOBT); 75 (FOBT+FS); 80 (COL) | Adherence rate; Test cost; Test sensitivity |
| Lejeune et al., France | FIT biennial | NR | NR | NR | Test cost |
| Lansdorp-Vogelaar et al., Netherlands | FIT biennial | NR | NR | 100 | Adherence rate |
| Pil et al., Belgium | FIT biennial | 2,351.67/QALY | 35,000/QALY | 42 (RS e COL)/60 (gFOBT) | Adherence rate COL (secondary); Test sensitivity (FIT); Test specificity (FIT); Sensitivity COL; Disease progression |
| Greuter et al., Netherlands | FIT biennial | Cost-saving | 35,916/LYG | 100 | Adherence rate; Test cost; Positive rate for small and larger adenomas |
| Lew et al., Australia | FIT biennial | 2,247.22/LYG | 50,000/LYG | 70 (FIT); 47 (COL) | CRC treatment cost; Confirmatory test cost (COL) |
| Areia et al., Portugal | FIT biennial | 4,892.94/QALY | 39,760/QALY | 65 | Adherence rate; Test cost; Probabilities related to CRC stages |
| Shimbo et al., Japan | FIT biennial | 17,884.83/LYG | NR | 45 | Adherence rate; Age screening started |
| Lew et al., Australia | FIT biennial | 2,115.69/LYG | 50,000/LYG | NR | No impacting variable |
| Barré et al., France | FIT biennial | 4,858.30/QALY | 40,000/QALY | 70 | Adherence rate; Test sensitivity (FIT); Test specificity (FIT) |
| Berchi et al., France | FIT biennial | 5,690.84/LYG | NR | NR | Adherence rate; Confirmatory test cost (COL) |
| Parekh et al., USA | FIT annual | 5,631.33/LYG | 50,000‒100,000/LYG | 60 | Adherence rate |
| Telford et al., Canada | FIT annual | 565.03/QALY | 50,000/QALY | 30 (FS); 42 (FIT); 19 (FS+FIT) | Adherence rate; Test sensitivity; Test cost; CRC treatment cost |
| Heitman et al., Canada | FIT annual | Cost-saving | NR | 100 | Adherence rate; Administrative costs (nonspecified) |
| Sharaf et al., USA | FIT annual | Cost-saving | 100,000/QALY | 53 (FOBT) e 39 (FS) | Adherence rate; Test cost; CRC treatment cost; CRC incidence |
| Barouni et al., Iran | FIT annual | 766.79/QALY | 50,000/QALY | 100 | No impacting variable |
| Ladabaum et al., Germany | FIT annual | Cost-saving | 25,000‒50,000/QALY | 100 | Adherence rate |
| Goede et al., Canada | FIT annual | Cost-saving | 50,000/QALY | 65 (first round)/ 80 subsequently rounds | Confirmatory test cost (COL); CRC treatment cost |
| Espinola et al., Argentina | FIT annual | 122.60/QALY | 102,856,50/QALY | 100 | Confirmatory test cost (COL); CRC treatment cost |
| Ladabaum et al., USA | FIT annual | Cost-saving | 100,000/QALY | 100 | Screening frequency (Stool DNA) |
| Hasdeu et al., Argentina | FIT annual | 1,257.82/LYG | 3 times GDP/per capita | Variable | NR |
| Murphy et al., United Kingdom | FIT annual | Cost-saving | 20,000/QALY | 40 | CRC treatment cost; Test sensitivity (FIT) |
| Jahn et al., Austria | FIT annual | 20,521.17/LYG | 15,000/LYG | 73 | Adherence rate; Test sensitivity (FOBT); Discount rate |
| Phisalprapa et al., Thailand | FIT annual | 688.86/QALY | 4,706/QALY | NR | No impacting variable |
| Hassan et al., France | FIT annual | 72,625.59/LYG | 50,000/LYG | 90 | Adherence rate |
| van Rossum et al., Netherlands | FIT annual | Cost-saving | NR | NR | Discount rate |
| van Ballegooijen et al., USA | FIT annual | 15,640.41/LYG | NR | 100 | Adherence rate; Test specificity |
| Dinh et al., USA | FIT annual (from 50) + COL only once at age 66 | Cost-saving | NR | 100 | No impacting variable |
| Arrospide et al., Basque country | FIT biennial | Cost-saving | NR | NR | No impacting variable |
| Ladabaum et al., USA | FIT annual | Cost-saving | 50,000/LYG | 100 | Adherence rate; Test sensitivity; Test cost |
| Knudsen et al., USA | FIT annual; gFOBT annual; FS 5-yearly; gFOBT annual + FS 5-yearly; FIT annual + FS 5-yearly; COL 10-yearly | Cost-saving | NR | 100 | Adherence rate; Test cost |
| O'Leary et al., Australia | FS 10-yearly | 18,267.38/LYG | NR | 60 | Adherence rate; Test cost |
| Regge et al., USA | FS 10-yearly | 10,768.40/LYG; 15,323.63/QALY | 100,000/LYG | 100 | Test sensitivity |
| Khandker et al., USA | FS 5-yearly | 19,403.15/LYG | NR | 90 | Adherence rate; Test sensitivity |
| Senore et al., Italy | FS once at age 58 | Cost-saving | 50,000/LYG | 100 | Adherence rate; Sensitivity test (FIT) |
| Sharp et al., Ireland | FS only once | 845.75/QALY | NR | 60 | Discount rate; Test cost; CRC treatment cost |
| van der Meulen et al., Netherlands | CT 3-yearly | 20,118.13/QALY | 20,000/QALY | Stool based: 60; Imaging: 40 | Adherence rate; Test cost; Inclusion of extra colonic findings |
| Chauvin et al., France | CT 5-yearly | 13,145.52/LYG | NR | 100 | Adherence rate; Discount rate; Time horizon |

COL, Colonoscopy; CT, Computed Tomography; FIT, Fecal Immunochemical Test; FOBT, Fecal Occult Blood Test; FS, Flexible Sigmoidoscopy; GDP, Gross Domestic Product; gFOBT, Guaiac Fecal Occult Blood Test; LYG, Life Years Gained; MRI, Magnetic Resonance Imaging; NR, Not Reported; QALY, Quality Adjusted Life Year; CET, Cost-Effectiveness Threshold.

^a^ All ICERs were adjusted by local inflation to 2020 and then converted into International dollars (I$) using the Organization for Economic Co-operation and Development (OECD) Purchasing Power Parity conversion rates. If the cost reference year was not reported, the year of publication was used as a reference for adjustments.
